# Supplementary material for: Exploring the phase diagram of the two-impurity Kondo problem
Source: Nat Commun. 2015 Nov 30;6:10046. doi: 10.1038/ncomms10046 (PMC4674668; doi:10.1038/ncomms10046)
Supplement: Supplementary Information — Supplementary Figure 1, Supplementary Table 1, Supplementary Note 1 and Supplementary References. [file ncomms10046-s1.pdf]

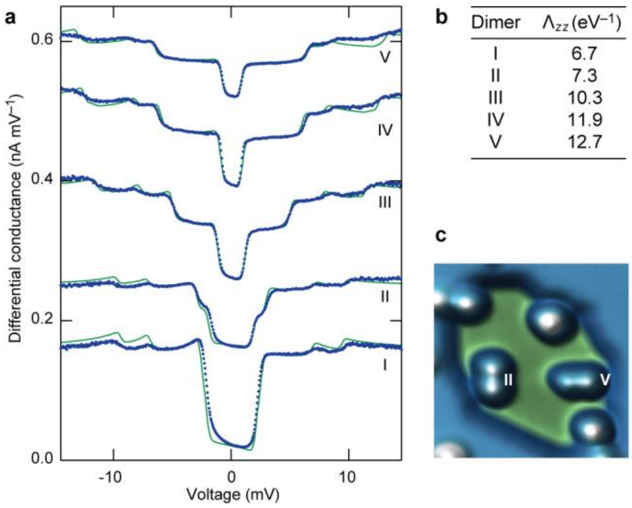

Supplementary Figure 1: Anisotropy variation in strained dimers. (**a**) Zero-field differential conductance spectra (blue dots) and corresponding simulated curves (green lines) recorded on different instances of a {2,0} dimer, showing variation in the spectroscopic features. (**b**) Best fit values for Λ_{zz} for all dimers shown in **a**; we found Λ_{xx}=0, Λ_{yy}=6.2 eV^{-1}, J=2 meV and $J_K\rho_S$=0.15 for all measured curves. (**c**) Topographic STM image of dimers II and V. These data also published in Ref. [1].

	$J_K\rho_s$	Λ_{xx} (eV^{-1})	Λ_{yy} (eV^{-1})	Λ_{zz} (eV^{-1})	J (meV)	D (meV)	E (meV)	g_x	g_y	g_z
single Co	0.15	0	6.4	6.4	–	2.8	0	2.0	2.3	2.3
{3/2, 3/2}										
atom 1 (marked)	0.15	0	6.3	6.3	0.12	2.8	0	2.0	2.3	2.3
atom 2	0.15	0	6.4	6.2	0.12	2.8	0.04	2.0	2.3	2.3
{0,2}	0.15	0	6.1	6.1	–0.22	2.7	0	2.0	2.3	2.3
{3/2, 1/2}										
atom 1 (marked)	0.15	0	6.2	6.4	–0.35	2.8	0.04	2.0	2.3	2.3
atom 2	0.15	0	7.0	7.0	–0.35	3.1	0	2.0	2.3	2.3
{1,1}	0.15	0	6.1	6.4	0.8	2.8	0.07	2.0	2.3	2.3
{2,0}	0.15	0	6.2	11.2	–2.0	3.8	1.1	2.0	2.3	2.5

Supplementary Table 1: Fitting parameter values. Parameter values used for the calculated curves in Fig. 3a-f. Atoms in dimers with inequivalent atom sites ({3/2,3/2} and {3/2, 1/2}) are marked in Fig. 3 with a red arrow. Parameters D, E, g_x, g_y and g_z are calculated from Λ_{xx}, Λ_{yy} and Λ_{zz} and are therefore not independent.

Supplementary Note 1: Magnetic anisotropy

Within each type of dimer except for the {2,0}, extracted anisotropy parameters were found to be consistent within ±5%, which is similar to the typical variation found in individual Co atoms. This variation may be attributed to local strain caused by subsurface defects [2]. However, as shown in Fig. S1, the {2,0} dimer was found to present much larger variations in the spectra. Interestingly, we could reproduce all the measured curves by varying only one fitting parameter, the anisotropy along the nitrogen direction Λ_{zz}, and keeping all the other parameters constant. We note that in the conventional notation of *D* and *E*, two parameters would have to be varied in order to model the observed behaviour. No correlation was found between the Λ_{zz} values and either the distance of the dimer from the edge of the Cu_2N island, or the size of the supporting island. In Fig. S1c two examples of {2,0} dimers are shown, that exhibit two extreme values of Λ_{zz}, but that were built on the same island and at very similar distances from the edges.

The large variation in the anisotropy of the {2,0} dimer can be accounted for since the lattice distortion is in a critical regime. It was noted for Fe dimers in this configuration [3] that the anisotropy is increased compared to the single atom case, due to an increase in the N-Co-N angle. For Co {2,0} dimers this angle approaches 180°: at this critical angle, the orbital levels become degenerate. Since Λ_{zz} is inversely proportional to the orbital splitting, close to the critical angle small strain-induced modifications of the crystal field can give rise to major changes in the anisotropy parameters. While the atoms in all other dimers are described as easy-plane systems with the hard axis along the vacancy direction (i.e. $\Lambda_{xx} < \Lambda_{yy} \sim \Lambda_{zz}$), for some instances of the {2,0} dimer Λ_{zz} has increased to such an extent that $\Lambda_{zz} - \Lambda_{yy} > \Lambda_{yy} - \Lambda_{xx}$, which classifies as an easy-axis system along the nitrogen direction. When written in terms of conventional phenomenological anisotropy parameters *D* and *E*, technically dimer IV should be specified as *D*=+3.99 meV, *E*=1.26 meV, and dimer V as *D*=-4.23 meV, *E*=1.37 meV. The smooth variation in the spectroscopy of Fig. S1a illustrates that it is more natural to use the description in terms of $\Lambda_{\mu\mu}$.

SUPPLEMENTARY REFERENCES

[1] Bryant, B. *et al*. Controlled complete suppression of single-atom inelastic spin and orbital cotunnelling. *Nano Letters* DOI 10.1021/acs.nanolett.5b02200 (2015).

[2] Otte, A. F. *et al*. The role of magnetic anisotropy in the Kondo effect. *Nature Physics* **4**, 847-850 (2008).

[3] Bryant, B., Spinelli, A., Wagenaar, J. J. T., Gerrits, M. & Otte, A. F. Local control of single atom magneto-crystalline anisotropy. *Physical Review Letters* **111**, 127203 (2013).
